# Inhaled Insulin Decoded: Dispelling Myths and Presenting Clinical Evidence

**DOI:** 10.7759/cureus.100247

**Published:** 2025-12-28

**Authors:** Jothydev Kesavadev, Hemant P Thacker, Neeta Deshpande, Arthur Asirvatham, Bharat Saboo, Pramila Kalra, Shehla Shaikh, Ameya Joshi, Manoj Chawla, Sanjay Agarwal, Jayashree Gopal, Abhijit Bhograj, Piya Ballani Thakkar, Rahul Iyer, Senthilnathan Mohanasundaram, Amarnath Sugumaran, Ashwin Karuppan, Lakshmi Nagendra

**Affiliations:** 1 Diabetes and Endocrinology, Jothydev’s Diabetes &amp; Research Center, Thiruvananthapuram, IND; 2 Cardiometabolic Medicine, Bhatia Hospital, Mumbai, IND; 3 Cardiometabolic Medicine, Jaslok Hospital, Mumbai, IND; 4 Diabetology, CentraCare Super Speciality Hospital &amp; Medical Research Centre, Belgaum, IND; 5 Diabetology, Arthur Asirvatham Hospital, Madurai, IND; 6 Diabetology, Prayas Diabetes Center, Indore, IND; 7 Endocrinology &amp; Metabolism, Ramaiah Medical College and Memorial Hospital, Bengaluru, IND; 8 Diabetology &amp; Endocrinology, Saifee Hospital, Mumbai, IND; 9 Endocrinology, Bhaktivedanta Hospital, Mumbai, IND; 10 Diabetes and Endocrinology, Lina Diabetes Care and Mumbai Diabetes Research Centre, Mumbai, IND; 11 Medicine, Aegle Clinic-Diabetes Care, Pune, IND; 12 Endocrinology and Diabetes, DiabEndoIndia, Institute for Diabetes, Endocrinology, Lifestyle and Metabolic Health, Chennai, IND; 13 Endocrinology, Manipal Hospital, Bengaluru, IND; 14 Endocrinology, Bombay Hospital and Medical Research Centre, Mumbai, IND; 15 Medical Affairs, Cipla Ltd., Mumbai, IND; 16 Internal Medicine and Diabetology, Gleneagles Global Health City, Chennai, IND; 17 Endocrinology, Jagadguru Sri Shivarathreeshwara (JSS) Medical College, Mysuru, IND

**Keywords:** afrezza®, inhaled insulin, inhaled therapy, lungs, myths, technosphere

## Abstract

Despite advancements in prandial insulin therapeutics, real-world usage of these products is associated with challenges such as injection-related anxiety, an increased risk of hypoglycemia, and weight gain. Other factors contributing to insulin non-adherence include travel-related storage issues, public embarrassment related to administration, and busy schedules. In India, where postprandial hyperglycemia remains a concern, there is a need for effective and patient-friendly strategies to manage glycemia adequately. Afrezza® (MannKind Corporation, Danbury, CT, USA), an inhaled Technosphere® insulin, provides a non-invasive, needle-free approach that has a rapid onset of action and short duration of action and has been demonstrated to closely mimic the physiological insulin profile. Afrezza® is a dry powder recombinant human insulin formulation adsorbed onto a proprietary carrier, developed for the delivery of insulin deep into the alveoli of the lungs, where it is absorbed into the systemic circulation. This review seeks to clarify the clinical role of inhaled insulin by addressing common myths associated with its usage and presenting evidence-based insights on its pharmacokinetic (PK) and pharmacodynamic (PD) profile, efficacy and safety, pulmonary safety, dosing considerations, storage requirements, use in special populations, and contraindications. The review also discusses practical aspects of Afrezza® use, especially proper patient screening based on spirometry testing, patient education, and counseling. Ultimately, Afrezza® signifies a transformation in insulin therapy, offering an effective and convenient alternative to prandial insulin for adult patients with type 1 diabetes mellitus (T1DM) or type 2 diabetes mellitus (T2DM).

## Introduction and background

Diabetes is a chronic metabolic disorder that arises from the body's inadequate production or ineffective utilization of insulin and is a leading cause of morbidity and mortality across the world [1,2]. The majority of individuals with diabetes have either type 1 diabetes mellitus (T1DM), resulting from the beta (β)-cell destruction, or type 2 diabetes mellitus (T2DM), resulting from a combination of defects in insulin secretion and insulin resistance [3]. As of now, an estimated 589 million adults aged 20-79 years, or one in nine globally, are living with diabetes. This number is projected to rise sharply to 853 million by 2050, highlighting the urgency of this growing global health challenge [4]. According to the International Diabetes Federation (IDF), the Southeast Asian region carries a significant diabetes burden. In India alone, with an adult population of approximately 947 million in 2024, the diabetes prevalence rate is 10.5%, amounting to around 89.8 million adults affected by the condition [5].

Physiological insulin replacement forms a cornerstone of treatment for patients with T1DM and those with advanced T2DM [6]. Many patients with T2DM need insulin therapy due to the progressive nature of the disease [3]. Basal insulin is the initial step in insulin therapy, helping to control fasting glucose, but it often fails to manage postprandial glucose (PPG). When glycemic targets are not met, incorporating selective PPG targeting treatments such as mealtime insulin, dipeptidyl peptidase inhibitors, thiazolidinediones, α-glucosidase inhibitors, and short-acting glucagon-like peptide-1 receptor agonists can help [7]. Controlling PPG is challenging [8] due to factors like meal composition, timing, and a mismatch between glucose absorption and insulin action. Patient behaviors, such as misestimating carbohydrate intake, can negatively affect glycemic control. Additionally, delaying or skipping insulin may further worsen blood sugar management. Rapid-acting insulin analogs need to be administered approximately 15 to 20 minutes before the start of a meal to best meet postprandial insulin needs [9]. Regular human insulin, which has a slower onset of action compared to rapid-acting analogs, typically requires administration 30 to 40 minutes before meals [10,11]. A study by Lane et al. indicated that various challenges are associated with mealtime insulin use, such as mismatched timing and amount of food intake, fear of hypoglycemia, forgotten doses, dosing errors, injection pain, and embarrassment [8]. 

Both patients and healthcare providers identified significant reasons for insulin non-adherence, including skipped meals, travel, public embarrassment, and busy schedules. Physicians also noted delays in the initiation of insulin and dose adjustments. This highlights the need for improved strategies to enhance adherence and outcomes [12].

Afrezza® (MannKind Corporation, Danbury, CT, USA), inhaled Technosphere® insulin, offers a novel, non-invasive approach that could overcome several barriers to initiating insulin therapy [3]. Unlike traditional injectable rapid-acting insulins, Afrezza® utilizes Technosphere® particles that deliver insulin via pulmonary absorption, resulting in an ultra-rapid onset and a shorter duration of action. This profile more closely mimics the physiological first-phase insulin response and may reduce the need for pre-meal timing precision [13,14]. Afrezza® is an ultra-rapid-acting insulin administered with an oral inhaler and approved for improving glycemic control in adults with diabetes mellitus (DM) [3]. Its approval indicates advancements in addressing the unmet needs of the patients who are unable or reluctant to use injectable prandial insulin. Inhaled insulin is often misunderstood due to myths surrounding its ease of use, safety, and effectiveness. Demystifying these myths could help healthcare professionals and patients consider inhaled insulin as a viable and convenient approach to insulin therapy [15].

This review aims to clarify the clinical role of inhaled insulin by dispelling common misconceptions and providing evidence-based insights into its pharmacokinetic (PK) and pharmacodynamic (PD) properties, pulmonary safety, dosing considerations, storage requirements, use in special populations, and contraindications. Additionally, it highlights practical aspects of Afrezza® use, including appropriate patient selection, as well as the importance of patient education and counseling for optimal outcomes.

## Review

Myth demystification

Myth 1: Lungs Are Not a Viable Route for Insulin Delivery

Fact: Lungs are a proven and effective route for rapid insulin delivery. Lungs possess approximately 500 million alveoli and unique features, including a high surface area, good vascularization, an enormous capacity for solute exchange, and an ultra-thin alveolar epithelium, which enables rapid absorption in pulmonary drug delivery. Lungs lack insulin-degrading peptidases present in the stomach [16,17]. Also, the first-pass metabolism of the gastrointestinal tract is avoided [16].

Inhaled insulins, stepping stones to Afrezza®: The quest for needle-free insulin is nearly as old as the origins of insulin, with the idea of administering insulin by breathing an aerosol being proposed as early as 1925 [18]. However, it took several years of research and development before the first inhaled insulin, Exubera®, was approved in 2006. Despite the breakthrough, initial efforts faced setbacks, and Exubera® was withdrawn from the market on October 18, 2007, due to its bulky inhaler device, restrictive prescribing guidelines (e.g., the National Institute for Health and Clinical Excellence (NICE) limiting its use only to those with needle phobia or needle site issues), milligram-based dosing (instead of unit-based dosing), concerns over the lack of long-term pulmonary safety data, and high cost along with low sales. These were major factors contributing to its withdrawal [19]. Currently, an inhaled insulin formulation utilizing a distinct technology and known as inhaled Technosphere® insulin (Afrezza®) received the U.S Food and Drug Administration (FDA) approval in 2014 and remains the only inhaled insulin option available for the management of diabetes [20]. It is an orally inhaled rapid-acting Technosphere® insulin administered via a thumb-sized, breath-powered inhaler and has continued to be available in the USA for more than 10 years. It addresses various shortcomings of the previously launched inhaled insulin with the small, sleek, convenient delivery system, unit-based dosing, and improved glycemic control in both T1DM and T2DM patients [21, 22]. Recently, the Central Drugs Standard Control Organization in India has approved Afrezza® in adults with DM [23].

Development of inhaled Technosphere® insulin (Afrezza®): Afrezza®, an orally inhaled insulin that addresses the shortcomings of earlier inhaled insulin formulations and provides a convenient therapeutic alternative to injectable insulins [24]. Developed by Alfred Mann [25], it is a dry powder form of recombinant human insulin adsorbed onto Technosphere® microparticles [24] made of fumaryl diketopiperazine (FDKP), which self-assembles into crystalline structures. Technosphere® microparticles include a median diameter of 2-2.5 µm and a similar aerodynamic diameter. Insulin is adsorbed onto these Technosphere® particles with a size suitable for drug absorption, resulting in Technosphere® insulin powder [18]. Upon inhalation, the particles reach into alveoli, and both insulin and FDKP are rapidly absorbed into the systemic circulation, reaching their maximum serum concentration within approximately 12-15 minutes [24,26], faster than that of subcutaneously administered insulin. The chemically inert excipient FDKP is not metabolized and is eliminated unchanged via the kidneys [25,27]. Afrezza® is approved for adults with T1DM and T2DM [25,28].

Hence, contrary to the myth, the lungs provide a viable and effective route for insulin delivery.

Myth 2: Afrezza® Has an Unpredictable PK/PD Profile Compared to Subcutaneous (SC) Insulin

Fact: Afrezza® offers a faster, more physiological PK/PD profile, ideal for rapid post-meal glucose control.

PK data: Inhaled Technosphere® insulin offers advantages, including the avoidance of injections. The unique physiological and anatomical features of the lungs contribute to rapid systemic absorption. The PK and PD data from various studies have suggested a time-action profile of inhaled insulin suitable for prandial use [17]. A study reported that inhaled Technosphere® insulin showed a time of maximum concentration (Tmax) of 12-17 minutes, and SC insulin showed a Tmax of 134 minutes. For inhaled Technosphere® insulin, doses of 25, 50, and 100 U are reported to have relative bioavailability of 25%, 23%, and 21%, respectively. Also, the maximum bioeffect was earlier for all three doses of inhaled Technosphere® insulin (42, 50, and 58 minutes) in comparison to SC insulin (171 minutes) [18]. Another prospective, controlled, open-label, randomized study with 13 people with T2DM reported that inhaled Technosphere® insulin produced a Tmax of ~17 min (vs ~135 min for SC regular human insulin [19]. Studies in both T1DM and T2DM patients revealed a quicker Cmax and glucose-lowering effects with inhaled Technosphere® insulin (10-15 minutes), suggesting a better prandial profile compared to the rapid-acting analogs [29].

PD data: Another study compared inhaled Technosphere® insulin with SC insulin in patients with T2DM and found that 48 units of inhaled Technosphere® insulin and 24 units of SC insulin achieved a similar three-hour insulin exposure (area under the curve from 0 to three hours (AUC₀-₃h): 55.8 vs. 60.0 nmol·min·L⁻¹). The maximum or peak serum concentration (Cmax) of inhaled Technosphere® insulin was 50% higher (858 vs 438 pmol/L; P=0.0001), and its Tmax was markedly shorter (17 vs. 135 min; P=0.0001) when compared with SC insulin. The PD studies concluded that most of the inhaled Technosphere® insulin’s glucose-lowering effect occurred within the first three hours, accounting for about 59%, unlike regular insulin, which delivered only about 27% of its effect in that period [19].

Rave et al., in their randomized crossover study in patients with T2DM, compared inhaled Technosphere® insulin with SC regular human insulin. Over a seven-day treatment period, separated by a two- to seven-day washout period, inhaled Technosphere® insulin demonstrated a higher peak serum insulin concentration at 15 minutes, while for regular insulin it was around 120 min. The glucose AUC₀-₂₄₀ for inhaled Technosphere® insulin was lower compared to the SC insulin (282.8 vs. 546.7 mmol·min/L; P=0.007). Total serum insulin exposure was similar among the groups. Inhaled Technosphere® insulin had a more rapid absorption and achieved higher peak insulin levels than SC insulin, with a similar insulin exposure [20]. The inhaled Technosphere® insulin has a PK/PD profile that more closely mimics physiologic mealtime insulin secretion compared to SC insulin. Its early peak concentration, ultra-rapid absorption (within 15 min), and fast onset of action (about two to three hours) mimic physiological postprandial endogenous insulin responses, which are typically absent in diabetes patients [21]. This unique profile underlines inhaled Technosphere® insulin’s ability to provide rapid PPG control while minimizing late postprandial insulin exposure.

**Figure 1:**
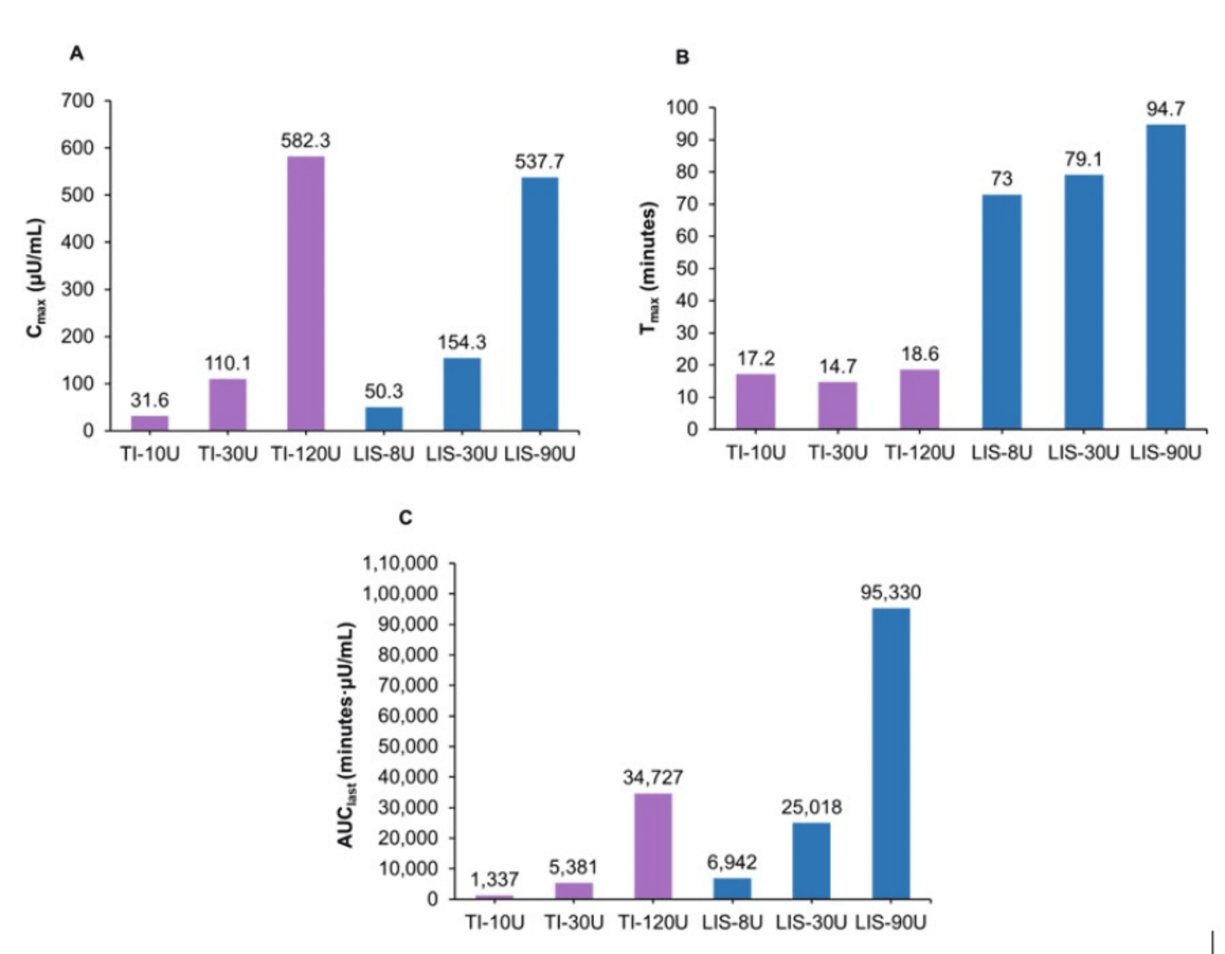
Summary of PK/PD parameters by dose Figure adapted from Grant M et al. Clin Pharmacokinet. 2022;61(3):413–22 [30]. This article is published under the Creative Commons Attribution License (CC BY 4.0). AUC: area under the curve; LIS: insulin lispro; PD: pharmacodynamic; PK: pharmacokinetic; TI: Technosphere® insulin; U: units.

A single-center randomized crossover trial compared the PK and PD of inhaled Technosphere® insulin with SC insulin lispro (LIS) in patients with T1DM. Patients received multiple doses of both insulins under euglycemic clamp conditions. The inhaled Technosphere® insulin showed a faster onset of action (seven to five minutes vs 21-38 minutes) and a shorter duration of action (two to six hours vs five to 10 hours) compared to LIS (Figure 1) [30].

These PK and PD studies demonstrate that inhaled Technosphere® insulin is associated with a shorter duration of action and better pharmacological activity compared to rapid-acting insulins.

Thus, inhaled Technosphere® insulin delivers a faster, more predictable PK/PD profile than SC insulin, making it well-suited for rapid post-meal glucose control.

Myth 3: Afrezza® Is Unsafe for Long-Term Use Because It Causes Serious Side Effects Like a Persistent Cough and Increases the Risk of Cancer

Fact: Afrezza® may cause a mild, transient cough in some users. Declines in forced expiratory volume in 1 second (FEV₁) are typically small, occur early, and are non-progressive; most resolve with discontinuation. Current data are insufficient to establish any association between inhaled Technosphere® insulin and malignancy. Overall, inhaled Technosphere® insulin has demonstrated consistent safety in long-term studies.

Cough: A mild, transient cough is the most commonly reported adverse effect. In pooled analyses of 13 phase 2/3 trials (5,505 patients), cough occurred in 28% of Afrezza® users versus 5.2% in comparators and was generally early, non-productive, and resolved over time [24]. In two double-blind, placebo-controlled studies in insulin-naïve T2DM patients, mild, transient dry cough occurred at similar rates with inhaled Technosphere® insulin (23.7% and 29.5%) and placebo (19.9% and 27.4%), suggesting the cough is related to the dry powder rather than the insulin itself. Discontinuation due to cough was low [21,25]. In a two-year study, cough was the most common respiratory event, typically mild, non-productive, occurring within 10 minutes of inhalation, appearing early, and declining over time [26].

**Figure 2:**
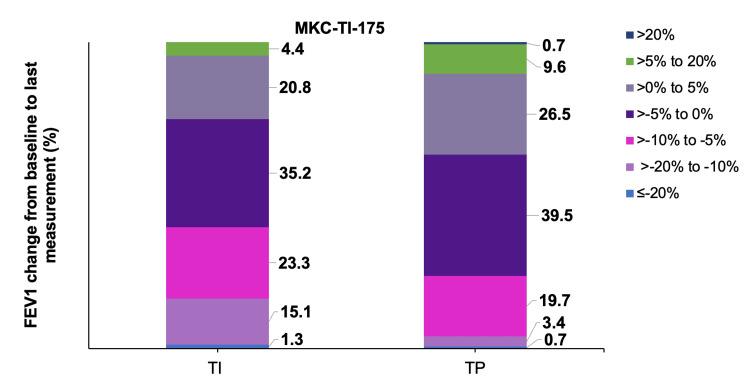
Distribution of percentage change in FEV1 from baseline to last measurement in the MKC-TI-175 a study (patients with T2DM) Figure adapted from McGill JB et al. Clin Drug Investig. 2020;40(10):973–983, published under the Creative Commons license. FEV_1: _forced expiratory volume in 1 second; T2DM: type 2 diabetes mellitus; TI: Technosphere® insulin; MKC-T1-175: MannKind Corporation Technosphere® Insulin

FEV_1 _decline: Patients treated with inhaled Technosphere® insulin experienced small and reversible declines in pulmonary function (measured by mean FEV_1_) from baseline compared with those receiving Technosphere® placebo or active comparators. Specifically, 35.2% and 23.3% of patients on inhaled Technosphere® insulin versus 39.5% and 19.7% of patients on placebo had FEV_1_ decline in the range of 0 to >-5% and -5% to >-10%, respectively (Figure 2) [24].

Small, non-progressive declines in FEV₁ were observed, which typically resolved within four weeks after discontinuation of therapy, regardless of treatment duration [24]. Routine spirometry is recommended at baseline, six months, and annually thereafter [27]. Notably, a case report by Parthvi et al. documented the resolution of pulmonary infiltrates attributed to inhaled Technosphere® insulin following cessation of the treatment [28]. Thus, long-term studies support inhaled Technosphere® insulin safety for chronic use.

Cancer: Long-term studies have shown no evidence of structural lung abnormalities on imaging, no nasal epithelial changes, lung pathology, or carcinogenicity associated with inhaled Technosphere® insulin [24,31,32].

In clinical trials, two cases of lung cancer occurred among patients treated with Afrezza® (two cases in 2,750 patient-years), both with a history of heavy tobacco use; no cases were reported in comparator groups (0 cases in 2,169 patient-years) [33].

Two additional post-trial cases were reported in non-smoking patients who had been exposed to Afrezza®, but available data are insufficient to determine causality. Population-level data suggest that inhaled insulin does not increase malignancy risk compared with the general diabetes population [26]. The carrier molecule FDKP shows no significant systemic accumulation, supporting long-term safety without dose adjustment [34]. A pooled analysis of 13 phase 2/3 clinical trials indicated similar rates of respiratory adverse events, pulmonary function changes, and lung malignancies between Afrezza®, active comparators, and placebo [24].

Overall, inhaled Technosphere® insulin is generally well tolerated, with mild side effects, transient cough, and minimal lung function changes. No clear link to cancer has been established based on current evidence.

Myth 4: Afrezza® Is Unsafe or Unsuitable for Use in Special Populations

Fact: Afrezza® has specific contraindications, but it can be a safe and effective option for many adult DM populations.

Patients with pulmonary disease: Afrezza® is contraindicated in patients with chronic lung disease, including asthma and chronic obstructive pulmonary disease (COPD), due to the risk of acute bronchospasm. Before initiating therapy, all patients should be evaluated with a medical history, physical examination, and spirometry (FEV₁) to identify underlying pulmonary disease. Acute bronchospasm has been reported in patients with asthma and COPD after Afrezza® dosing [35].

Pregnancy: In pregnant women with diabetes, inhaled Technosphere® insulin should only be used when the benefits outweigh the potential risks. Inhaled Technosphere® insulin has not yet been evaluated in pregnant women.

Lactation: The presence of Technosphere® insulin or its carrier in human breast milk has not been determined. There is a potential for transfer into breast milk; therefore, physicians should assess whether to discontinue nursing or medication.

Chronic kidney disease and chronic liver disease: The impact of inhaled Technosphere® insulin on patients with renal or hepatic impairment remains unknown. Hence, caution is needed when prescribing to patients with such comorbidities [36].

Geriatric use: In patients ≥65 years of age, clinical data have not shown any differences in effectiveness and safety in comparison to young adults.

Pediatric use: Inhaled Technosphere® insulin is not approved for use in individuals under 18 years of age due to the lack of clinical trials in this age group. A supplemental Biologics License Application for use in children and adolescents aged four to 17 years living with diabetes has recently been accepted for review by the U.S. FDA. Approval is pending, and clinical use in this age group remains off-label until a final regulatory decision is made [37].

The INHALE-1 study is a 26-week, open-label trial comparing inhaled Technosphere® insulin to multiple daily injections of rapid-acting insulin, both with basal insulin, in 230 patients with diabetes. The full intention to treat (ITT) analysis exceeded the 0.4% non-inferiority margin (0.435%) due to a non-adherent patient; the modified ITT analysis (excluding that patient) met the criteria (0.370%), thereby establishing the non-inferiority of inhaled Technosphere® insulin to multiple daily injections. Lung function remained stable in both groups, and no significant safety concerns or differences in hypoglycemia were observed [38].

With proper use, inhaled Technosphere® insulin can be safe and effective for many adults with diabetes, despite certain contraindications.

Myth 5: Afrezza® Inhalers and Cartridges Require Complicated Storage and Are Difficult to Manage

Fact: Both the Afrezza® inhaler and cartridges have simple, user-friendly storage requirements designed for convenience.

**Figure 3:**
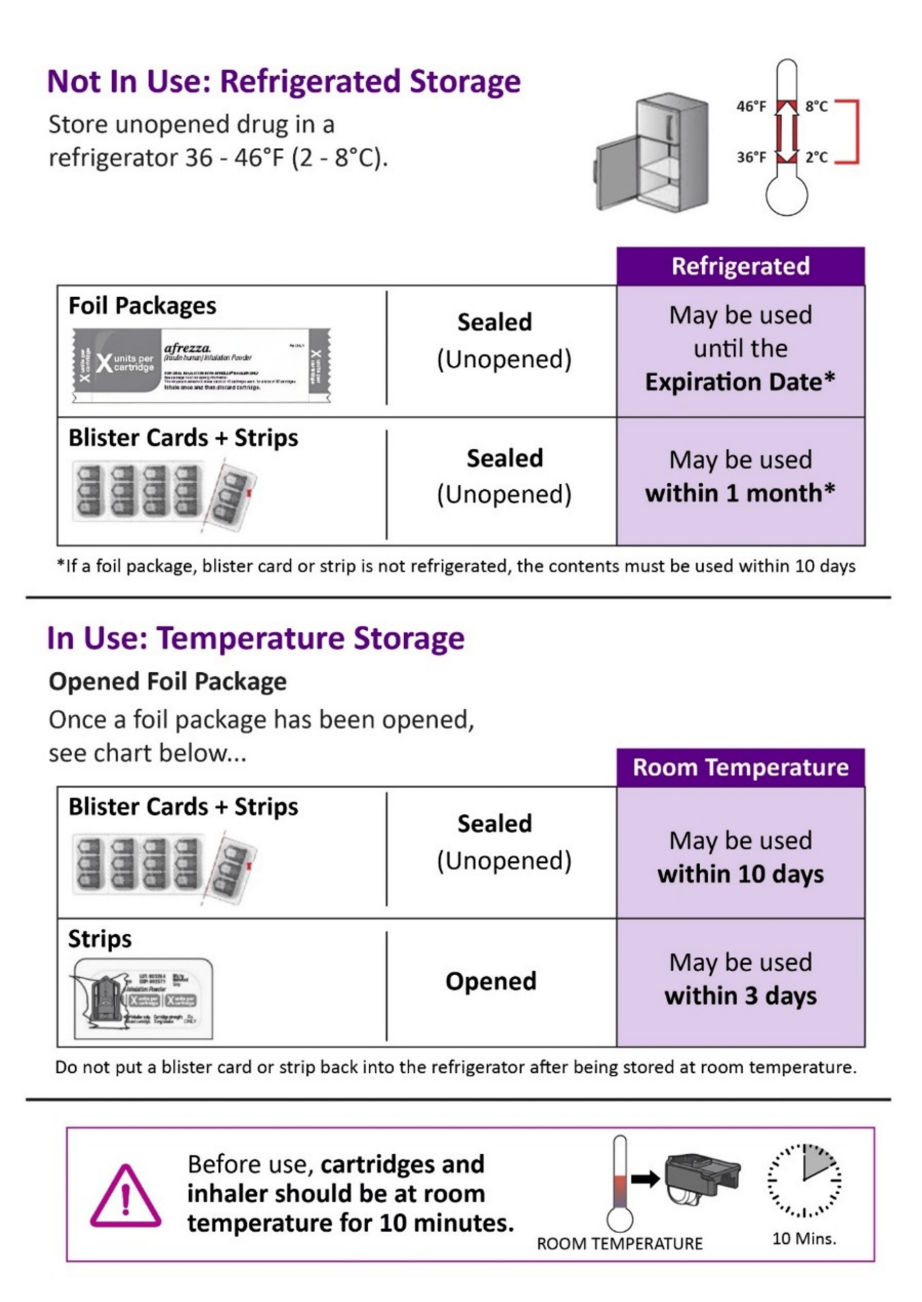
Storage of an Technosphere® insulin Figure adapted from [35].

Storage of insulin cartridges: Foil-wrapped unopened or sealed inhaled Technosphere® insulin cartridges could be stored in refrigerated conditions at 2-8°C and may be used until the expiration date. An unrefrigerated blister pack must be used within 10 days. After opening the package at room temperature, sealed strips or blister cards must be used within 10 days, and opened strips within three days. Cartridges should be allowed to sit out for 10 minutes at room temperature before use (Figure 3) [33,36,35].

Storage of the inhaler device: The inhaler device must be stored at 2°C to 25°C with brief excursions allowed. The inhaler should be allowed to reach room temperature for 10 minutes before use. One inhaler could be used with all the cartridge strengths and should be replaced every 15 days [34,36]. Thus, inhaled Technosphere® insulin offers simple and convenient storage, making it easy to manage.

Myth 6: Afrezza® Is Not As Effective as SC Insulin

Fact: Afrezza® is as effective as subcutaneously injected insulin in patients with diabetes.

Afrezza® is as effective as SC insulin in glycemic control [36]. Clinical trials demonstrate non-inferiority to insulin aspart (IA) in glycated hemoglobin (HbA1c) reduction, with a faster onset of action and reduced risk of hypoglycemia.

A 24-week trial compared HbA1c changes in patients with T1DM receiving either inhaled Technosphere® insulin or SC IA, both administered with basal insulin. The mean HbA1c reduction was -0.21% with inhaled Technosphere® insulin (from a baseline of 7.94%) and -0.40% with IA. The between-group difference was 0.19% (95% CI 0.02-0.36), which satisfied the predefined noninferiority criterion (<0.4%). The most common side effect of inhaled Technosphere® insulin was a cough (generally mild; 31.6% vs. 2.3% with aspart). Hypoglycemia occurred in 96.0% of patients in the inhaled Technosphere® insulin group and 99.4% in the aspart patients, with no statistical significance (P=0.0621). However, the event rate was significantly lower with inhaled Technosphere® insulin than with aspart patients (9.8 vs. 14 events per patient-month; (P<0.0001)). The study concluded that inhaled Technosphere®insulin was non-inferior to aspart in reducing HbA1c, with the added benefit of less hypoglycemia, but with a higher incidence of cough [39]. Similarly, in another 24-week randomized trial comparing inhaled Technosphere® insulin and IA added to basal insulin in adults with T2DM, both treatments significantly reduced HbA1c levels (inhaled Technosphere® insulin: -1.0%; IA: -1.3%). Inhaled Technosphere® insulin was associated with a modest weight loss (-0.78 kg), while IA led to a slight weight gain (+0.23 kg). Inhaled Technosphere® insulin had fewer subjects reporting ≥1 hypoglycemic event (43%) compared to IA (54%) (P=0.035). The event rate (events/subject/month) was also lower for inhaled Technosphere® insulin (0.65) than for IA (0.97). Overall, inhaled Technosphere® insulin showed comparable glycemic efficacy to IA with a lower risk of hypoglycemia [40]. Thus, inhaled Technosphere® insulin is as effective as SC insulin for managing diabetes.

Myth 7: Afrezza® Is the Same as Exubera®

Fact: Afrezza® has a distinct profile vs. Exubera® in terms of product characterisation, pharmacology, efficacy, and safety.

Exubera® was the first inhaled insulin product approved by the FDA in 2006, but it failed due to its bulky device type, cumbersome device usage procedure, complex dosing, and several other reasons mentioned earlier in the article. The inhaled Technosphere® insulin, which received FDA approval a decade ago, offers potential improvements by providing a user-friendly, smaller device with unit-based doses, demonstrating a good balance of efficacy and safety. The inhaled Technosphere® insulin design, better PK profile, and user friendliness indicate a marked advancement over previous inhaled insulins [38]. Exubera® was a recombinant DNA-derived, spray-dried powder containing insulin and other excipients like glycine, mannitol, and sodium citrate. The particle size of the inhalation formulation was ~3 µm, optimized for deep lung deposition [41]. In contrast, inhaled Technosphere® insulin is a dry recombinant human insulin powder adsorbed onto FDKP. It has a particle size of 2-2.5 µm, which allows deep lung delivery. It is delivered via a small inhaler device [42]. Overall, inhaled Technosphere® insulin represents a significant evolution in inhaled insulin therapy, addressing many of the drawbacks that led to Exubera®'s failure. Its compact inhaler device design, simple dosing, and favourable PK profile improve both patient acceptance and clinical utility, positioning it as a more effective and practical alternative for mealtime insulin delivery. Thus, inhaled Technosphere® insulin is a distinct and more advanced inhaled insulin compared to Exubera®, with differences in efficacy, safety, and pharmacology.

Myth 8: Afrezza® Eliminates the Need for All Insulin Injections

Fact: Afrezza® only replaces mealtime (prandial) insulin and not basal insulin.

Inhaled Technosphere® insulin acts as an add-on therapy for patients on long-acting insulin in T1DM, and it may be used alongside oral antihyperglycemic agents or insulin in patients with T2DM who are unable to meet glycemic targets [43]. Thus, inhaled Technosphere® insulin replaces only mealtime insulin, not basal injections.

Myth 9: Afrezza® Was Launched and Then Withdrawn

Fact: Afrezza® has been launched and has been available for more than 10 years

Contrary to the misconception, inhaled Technosphere® insulin was never withdrawn. An inhaled Technosphere® insulin product was approved by the FDA in 2014 and has remained available for more than 10 years. Its safety and efficacy have been demonstrated in multiple trials in both T1DM and T2DM. While long-term data are not available, it is an approved therapy backed by real-world clinical evidence. Thus, inhaled Technosphere® insulin has remained available for over a decade and was never withdrawn.

Clinical use recommendations for inhaled Technosphere® insulin in real-world settings

Inhaled Technosphere® insulin is approved for use in adults with T1DM and T2DM for diabetes control. The inhaled Technosphere® insulin offers the benefit of convenience and faster action in insulin therapy. Before starting treatment with inhaled Technosphere® insulin, the clinicians must record medical history, physical examination, and spirometry analysis. The inhaled Technosphere® insulin is prescribed for adult patients (18 years old or more) at the start of the meal, utilizing one inhalation per cartridge. A simple guide for dose conversion during transition from SC insulin to inhaled Afrezza®: a dose of up to four units of SC insulin corresponds to four units of Afrezza®, five to eight units to eight units, nine to 12 units to 12 units, 13 to 16 units to 16 units, 17 to 20 units to 20 units, and 21 to 24 units to 24 units [36]. Further, dosing recommendations for inhaled Technosphere® insulin are depicted in Table 1 [36].

**Table 1:** Dosing recommendation Table adapted from Goldberg T, PharmD, BCPS, and Wong E, PharmD, BCPS. Afrezza® (Insulin Human) Inhalation Powder: A New Inhaled Insulin for the Management of Type 1 or Type 2 Diabetes Mellitus. Drug Forecast. 2016;11:735–741 [36]. SC: subcutaneous

Patient type	Recommended dose
Insulin-naive	Start with four units of inhaled Technosphere^®^ insulin at each meal
On SC insulin	The dose conversion chart is used to determine the appropriate inhaled Technosphere^®^ insulin dose at every meal
On SC premixed insulin	Determine the total daily dose of premixed insulin and divide half of this dose equally into three meals a day. The estimated SC mealtime dose should then be converted to an appropriate inhaled Technosphere^®^ insulin dose. The remaining half of the total daily insulin dose should be given as a basal insulin dose.

Role of inhaled Technosphere® insulin in complementing existing insulin regimens

Inhaled Technosphere® insulin, a rapid-acting insulin, is approved for use in both T1DM and T2DM patients, intended to address prandial glucose excursions as discussed previously, and is not a replacement for basal insulin. It could be integrated into an existing insulin regimen for optimizing PPG control [44]. This is typically advantageous in patients who are hesitant to start or intensify insulin therapy owing to the injection concerns and may improve patient compliance [45]. The inhaled Technosphere® insulin also serves as an add-on therapy for patients on basal insulin and oral antidiabetic agents who are unable to meet glycemic goals due to post-meal glucose spikes [43]. Its quick onset and short duration of action tend to closely mimic physiological insulin, allowing for flexible dosing around the meals compared to the rapidly acting insulin analogs [13]. Thus, clinicians can confidently consider inhaled Technosphere® insulin for managing PPG in patients with T1DM and T2DM, and the benefits offered by inhaled Technosphere® insulin may potentially lead to better adherence, acceptance, and glycemic outcomes.

Efficacy of inhaled Technosphere® insulin is presented in Table 2.

**Table 2:** Efficacy of inhaled Technosphere® insulin CI: confidence interval; HbA1c: glycated hemoglobin; OHA: oral antihyperglycemic drugs.

Study design	Sample size (N)	Intervention	Outcomes	Author
Double-blind, placebo-controlled trial	353	Prandial inhaled Technosphere® added to existing OHAs in insulin-naïve adults with type 2 diabetes	HbA1c reduction at 24 weeks: −0.8% vs. −0.4%; 38% vs. 19% achieved HbA1c ≤7.0%; slight weight gain (+0.5 kg vs. −1.1 kg); mild transient cough; small reversible decline in lung function	[25]
Randomized, multicenter trial	518	Inhaled Technosphere® insulin vs. subcutaneous insulin aspart + basal insulin in patients with type 1 diabetes	HbA1c reduction at 24 weeks: −0.21% vs. −0.40% (insulin aspart); HbA1c <7.0% (18.3% vs. 30.7%); weight change: -0.4 kg vs. +0.9 kg (P=0.0102); fewer hypoglycemia events (9.8 vs. 14.0/month; P<0.0001)	[39]
Randomized controlled trial	309	Inhaled Technosphere® insulin + basal insulin (glargine) or Insulin aspart + basal insulin (glargine)	HbA1c change at 24 weeks: 8.9% to 7.9% vs. 9.0% to 7.7; treatment difference 0.26% (not significant below equivalence margin); weight change: −0.78 kg vs. +0.23 kg (P=0.0007); lower mild-moderate hypoglycemia risk	[40]
Phase III randomized, double-blind, placebo-controlled, parallel-group	216	Randomized (2:1) to inhaled Technosphere® insulin or placebo	HbA1c reduction at 12 weeks: 0.62% vs. 0.20%; treatment difference of 0.417% (P=0.0124)	[46]
Multicenter randomized controlled trial	213	Prandial inhaled Technosphere® insulin + basal insulin degludec in adults with type 1 diabetes	At 30 weeks: HbA1c reduction -0.21% from week 17 (P < 0.001); HbA1c <7.0% increased from 21% to 42%; time in range improved 52% to 54%	[47]

Safety profile of inhaled Technosphere® insulin

The inhaled Technosphere® insulin offers promising advantages in terms of feasibility, PK, and convenience. However, safety is always a crucial consideration for any therapy. In patients with T1DM, Afrezza® must be used with basal insulin. It is not recommended for the treatment of diabetic ketoacidosis [35]. The most common adverse reactions reported in clinical trials were hypoglycemia, cough, and throat pain or irritation [13].

Patient screening

In one randomized, multicenter trial, an inclusion criterion required that the participants show an FEV_1_ and forced vital capacity (FVC) of ≥70% of predicted before enrollment, underscoring the necessity of adequate baseline pulmonary function before the inhaled Technosphere® insulin therapy [39]. Spirometry is considered to be normal when the FEV_1_/FVC ratio exceeds 0.70, with both FVC and FEV_1_ higher than 80% of the predicted values [48]. It is important to note that before initiating inhaled Technosphere® insulin therapy, baseline spirometry FEV_1_ is required for all patients, followed by repeat assessments at six months and annually to identify potential lung disease. Furthermore, if there is a ≥20% decline in FEV_1_ from baseline, treatment discontinuation can be considered. Patients with respiratory symptoms (e.g., wheezing, cough, shortness of breath) may need more frequent monitoring, and persistent symptoms should prompt discontinuation of inhaled Technosphere® insulin [33].

Smoking status and other lung diseases

Smoking status is another important factor; inhaled Technosphere® insulin is not recommended in current smokers and those who have recently stopped smoking (less than six months). Inhaled Technosphere® insulin is not recommended in patients with chronic lung diseases such as asthma and COPD due to the risk of acute bronchospasm [33]. Clinical trials have demonstrated a favorable safety profile when used in properly screened patients with respect to lung health.

Key counseling points for the patients

Efficient patient education plays a crucial role in improving treatment adherence and optimizing therapeutic outcomes. When initiating therapy with inhaled Technosphere® insulin, it is essential to provide clear guidance on key practical aspects. Patients should be instructed on proper storage conditions and trained in the correct use of the inhaler, including how to load and handle the cartridges. It is also important to dispel common misconceptions, for example, by informing patients that inhaled Technosphere® insulin is designed to complement basal insulin therapy and is not limited to individuals with T1DM; it is also suitable for selected patients with T2DM. Dosing instructions must be communicated, emphasizing that inhaled Technosphere® insulin should be taken at the start of a meal for optimal glycemic control. Patients should be encouraged to promptly report any new respiratory symptoms or side effects. The need for regular lung function monitoring through spirometry should be explained, helping patients understand the importance of safety surveillance throughout treatment.

By addressing these practical considerations, clinicians can more confidently recommend inhaled Technosphere® insulin as a safe, patient-friendly, non-invasive, and innovative insulin option that is well-aligned with the evolving needs of diabetes care in India.

Limitations and future directions

Although evidence supporting inhaled insulin continues to grow, important limitations remain. Long-term data, especially in Indian populations, are limited, and cost and accessibility challenges may restrict wider use. Future studies should include pediatric cohorts, real-world effectiveness data, and evaluations of inhaled insulin as part of combination regimens to better define its clinical role.

## Conclusions

Inhaled Technosphere® insulin (Afrezza®) represents a transformative advancement in prandial insulin therapy, offering a viable alternative to traditional SC injections. With its rapid onset, shorter duration of action, and favorable safety profile, inhaled Technosphere® insulin addresses several long-standing challenges in mealtime insulin management, including injection burden, delayed action, and risk of late postprandial hypoglycemia. While skepticism surrounding previous inhaled insulin remains, the robust clinical evidence supporting inhaled Technosphere® insulin, combined with improved device design and clear usage guidelines, underscores its potential in appropriately selected patients. As India continues to witness a rising burden of diabetes, inhaled Technosphere® insulin holds promise in enhancing patient adherence and satisfaction through its ease of use and physiological insulin kinetics. However, its successful adoption hinges on raising awareness, dispelling misconceptions, and equipping healthcare professionals with the knowledge to identify suitable candidates through appropriate screening. Future studies in the Indian population will be instrumental in validating its role and maximizing its impact in real-world clinical settings.
